# Primary Uterine Rhabdomyosarcoma in a 54-Year-Old Postmenopausal Woman

**DOI:** 10.7759/cureus.9841

**Published:** 2020-08-18

**Authors:** Ala M Aljehani, Ahmed Abu-Zaid, Osama Alomar, Emad A Jabrah, Abdulmohsen Alkushi

**Affiliations:** 1 Medicine, Imam Mohammad Ibn Saud Islamic University, Riyadh, SAU; 2 Medicine, Alfaisal University, Riyadh, SAU; 3 Obstetrics and Gynecology, King Faisal Specialist Hospital and Research Centre, Riyadh, SAU; 4 Medicine, King Saud Bin Abdulaziz University for Health Sciences, Riyadh, SAU

**Keywords:** rhabdomyosarcoma, uterine, myogenic progenitor cells, adult

## Abstract

Rhabdomyosarcoma (RMS) is a malignant neoplasm that originates from undifferentiated myogenic progenitor cells. It is predominantly a pediatric disease, and its occurrence in adults is exceedingly rare. Adult primary RMS of gynecologic origin is an uncommon phenomenon, and the cervix is the most frequently involved site. The incidence of adult primary uterine RMS is extremely scarce. Herein, we present the case of primary uterine RMS in a 54-year-old Saudi postmenopausal woman who presented to clinic attention with a six-month history of abdominal pain and vaginal bleeding.

## Introduction

Rhabdomyosarcoma (RMS) is a malignant neoplasm that originates from undifferentiated myogenic progenitor cells [1]. It is largely a disease of childhood and is the most frequently diagnosed soft tissue tumor found in children, comprising up to nearly 50% of all pediatric soft tissue tumors [2]. RMS can originate anywhere in the body; however, the head and neck region is the most frequent site of RMS involvement in children [3]. Adult RMS is exceedingly uncommon, accounting for less than 4% of all soft tissue sarcomas specifically, and 1% of all malignancies generally [4,5]. Deep soft tissue of limbs is the most frequent site of involvement in adult RMS. Adult RMS involving the genitourinary system is exceedingly rare. Particularly, primary RMS of the uterine cavity is exceptionally scarce with roughly less than 35 reported cases in the English literature [3]. Histopathologically, uterine RMS is divided into three major categories, namely pleomorphic, embryonal and alveolar types [6].

Owing to the rarity of uterine RMS, diagnosis is often challenging and largely delayed [3]. Herein, we present a case of primary embryonal RMS of the uterus in a 54-year-old postmenopausal woman who presented to clinical attention with a six-month history of abdominal pain and vaginal bleeding.

## Case presentation

A 54-year-old Saudi postmenopausal woman presented to the clinic with a six-month history of abdominal pain and vaginal bleeding. The abdominal pain was mainly localized to the lower abdomen. The pain was dull in nature, continuous, non-radiating and moderately intense (6 out of 10 in terms of severity). The complaints were associated with shortness of breath, palpitations, loss of appetite and weight loss. The patient denied symptoms of urinary retention/incontinence, constipation or bleeding per rectum. Past medical history was significant for diabetes mellitus. Past surgical history and family history were unremarkable. Socially, she was a widowed housewife and non-smoker. With regard to the obstetric and gynecologic history, menarche started at 12 years of age. The menstrual cycles were regular. No use of oral contraceptive pills or hormonal replacement therapy for the past 10 years. Menopause occurred seven years ago. The last gynecological checkup was eight years ago, and Pap smear was negative. The patient had two uncomplicated pregnancies with two babies delivered vaginally at full term.

Laboratory testing revealed a blood hemoglobin level of 9.2 g/L (normal range: 11.9 to 15.1 g/L) and tumor markers were negative. On general examination, a mass protruding through the endocervical canal with active bleeding was seen.

**Figure 1:**
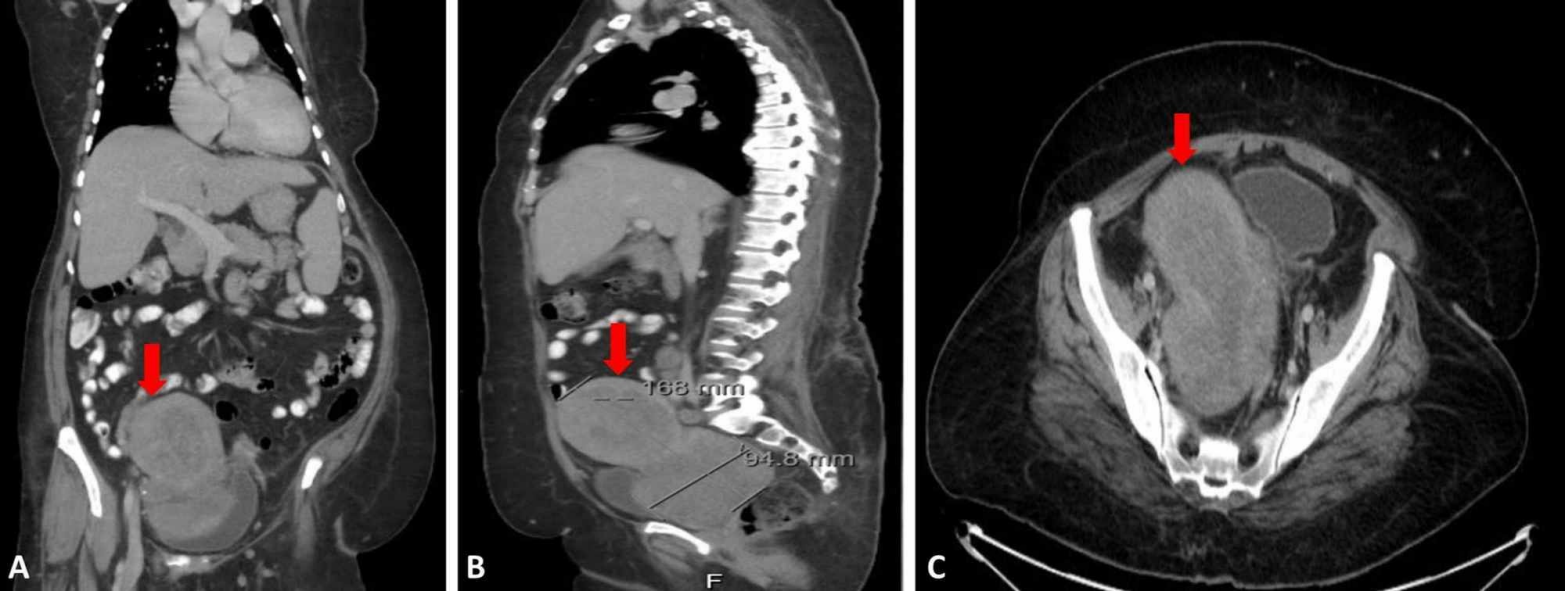
Coronal (A), sagittal (B) and axial (C) CT scans demonstrating a large uterine mass (red arrow) extending into the upper vagina and abutting the rectum and urinary bladder.

CT scan showed a large uterine soft tissue mass extending to the upper vagina and abutting the rectum and urinary bladder (Figure 1). There were multiple metastatic pelvic and lower paraaortic lymph nodes. Also, deep pelvic nodularity worrisome for early peritoneal carcinomatosis was observed. Additionally, several right pulmonary nodules were noted. 

**Figure 2:**
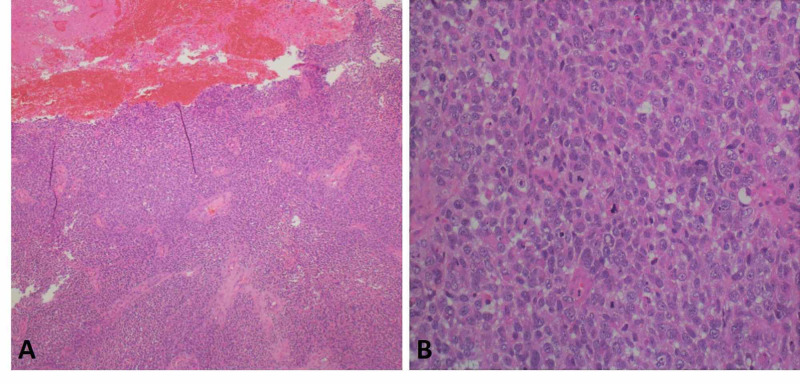
Microscopic examination of the protruding cervical biopsy showing sheets of malignant cells with hemorrhage and necrosis (A). The neoplastic blue cells were discohesive and had spindle-shaped morphology, frequent mitoses and bizarre nuclei (B).

A biopsy from the protruding cervical mass was obtained for histopathological analysis. The specimen consisted of multiple pieces and fragments of soft red-tan tissue measuring in aggregates 10 cm. Microscopic examination of the tissue showed sheets of malignant cells with hemorrhage and necrosis. The neoplastic blue cells were discohesive and had spindle-shaped morphology, frequent mitoses and bizarre nuclei (Figure 2).

**Figure 3:**
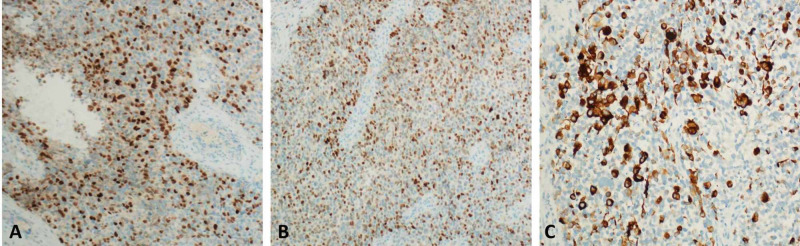
Immunohistochemical (IHC) staining of the protruding cervical biopsy showing diffuse positivity of the neoplastic cells to myogenin (A), MyoD1 (B) and desmin (C).

Immunohistochemical (IHC) staining showed diffuse positivity of the neoplastic cells to myogenin, MyoD1 and desmin (Figure 3). Conversely, the neoplastic cells showed negative staining for PAN-CK, P63 and CD99. Fluorescence in situ hybridization (FISH) analysis for Forkhead box protein O1 (FOXO1) gene rearrangement was not detected. In view of the clinical, radiological and histopathological investigations, the final diagnosis was consistent with primary uterine embryonal RMS.

The case was discussed in a multidisciplinary tumor board meeting. In consideration of the advanced stage disease presentation, the consensus was to consider palliative pelvic radiotherapy (a total dose of 30 Gy, divided into three fractions monthly) to control the active bleeding. The patient would be reassessed after three months. 

## Discussion

RMS is predominantly a pediatric disease, and its occurrence in adults is exceedingly rare [2,4,5]. Adult primary RMS of gynecologic origin is an uncommon phenomenon [6]. Cervix is the most frequently involved site in adult RMS of gynecologic origin [3]. With regard to uterus, less than 35 cases of primary uterine RMS have been reported in the English literature to date. To the best of our knowledge, we presented the first ever case of primary uterine RMS from Saudi Arabia.

Clinically, vaginal bleeding is the most frequent presenting symptom in patients with uterine RMS [3]. This finding was reciprocated in our patient too.

It is crucially vital to correctly differentiate between the three histopathological types of RMS as they hold substantial variances in biological behavior and prognosis [7]. Epidemiologically, for uterine RMSs, the most frequent histopathological type is pleomorphic RMS (60%-70%) and correlates with poor prognosis. Alveolar RMS is the least common (less than 5%), characterized genetically by FOXO1 chromosomal rearrangements and associated with unfavorable prognosis. Lastly, embryonal RMS accounts for 30%-40% of all uterine RMSs, characterized molecularly by Kirsten rat sarcoma viral (KRAS)/p53 mutations and correlates with good prognosis [7]. The embryonal RMS histopathological type is further subcategorized into Botyroid and spindle cell variants. In embryonal RMS, neoplastic cells are small, round, spindled or strap-shaped; some of which have eosinophilic cytoplasm found in a myxoid background.

Due to the rarity of uterine RMS especially in adults, other common neoplasms should be ruled out. These neoplasms comprise leiomyosarcoma, high-grade endometrial stromal sarcoma, adenosarcoma and carcinosarcoma [6]. The positive IHC for myogenin and MyoD1 and negative IHC for caldesmon and estrogen receptor can be helpful in confirming the diagnosis of RMS [3].

The prognosis of adult RMS generally and uterine RMS specifically is extremely unfortunate, with the vast majority of patients presenting with extensive disease at time of clinical diagnosis [3,8]. Our patient had multiple widespread metastases. The optimal therapy of adult patients with RMS of gynecologic origin is not defined. However, aggressive multimodality therapy comprising combination chemotherapy, radiotherapy and surgery, whenever technically feasible, yields better clinical outcomes [6].

Prognostic data about survival outcomes in adult patients with uterine RMS are very limited. This is largely ascribed to the small number of published cases about adult patients with uterine RMS [3]. However, Gerber and colleagues examined a total of 148 adult patients with RMS arising from gynecologic and non-gynecologic sites. The reported five-year overall survival (OS) rates for metastatic and non-metastatic patients were 45% and 26%, respectively [8]. Ferrari and colleagues explored the treatment outcomes in 171 adult patients with RMS originating from various sites. The reported chemotherapy response and five-year OS rates were 85% and 40%, respectively [9]. Ogilvie and colleagues inspected the multimodal (surgery, radiotherapy and chemotherapy) treatment outcomes in 11 adult patients with RMS. The reported two-year OS and disease-free survival (DFS) rates were 55% and 64%, respectively [10]. Little and colleagues scrutinized the multimodal (surgery, radiotherapy and chemotherapy) treatment outcomes in 82 adult patients with RMS. The 10-year OS and DFS rates were 40% and 41%, respectively [11].

## Conclusions

Uterine RMS in adults is a biologically aggressive malignancy with dismal prognosis despite the multimodal therapeutic strategies. Although rare, however, uterine RMS should be considered in the differential diagnosis of postmenopausal women presenting with vaginal bleeding. Histopathological examination in addition to immunohistochemistry and cytogenetic studies can aid in stabling the definitive diagnosis. Aggressive multimodality therapy yields better clinical outcomes.
